# Learning Nonlinear Dynamics of Flexible Structures for Predictive Control Using Gaussian Process NARX Models

**DOI:** 10.3390/biomimetics11040253

**Published:** 2026-04-07

**Authors:** Nasser Ayidh Alqahtani

**Affiliations:** Department of Mechanical Engineering, College of Engineering, Qassim University, Buraidah 51452, Saudi Arabia; n.alqahtani@qu.edu.sa

**Keywords:** nonlinear model predictive control, Gaussian Process Nonlinear AutoRegressive model with eXogenous input, predictive functional control, advanced process control

## Abstract

Biological systems regulate motion and suppress unwanted vibrations through learning, adaptation, and predictive control under uncertainty. Inspired by these principles, Bayesian system identification has emerged as a powerful framework for modeling and estimation, particularly in the presence of uncertainty in structural systems. Flexible structures in aerospace and robotics require advanced control to mitigate vibrations under model uncertainty. This paper proposes a data-driven strategy leveraging a Gaussian Process (GP) integrated within a Nonlinear Model Predictive Control (NMPC) framework. The core innovation lies in using a Gaussian Process Nonlinear AutoRegressive model with eXogenous input (GP-NARX) as a probabilistic predictor to capture structural dynamics while quantifying uncertainty. The operational mechanism involves a tight coupling where the GP provides multi-step-ahead forecasts that the NMPC optimizer uses to minimize a cost function subject to constraints. Validated through simulations on Duffing oscillators, linear oscillators, and cantilever beams, the GP-NMPC achieved an 88.2% reduction in displacement amplitude compared to uncontrolled systems. Quantitative analysis shows high predictive accuracy, with a Root Mean Square Error (RMSE) of 0.0031 and a Standardized Mean-Squared Error (SMSE) below 0.05. Furthermore, Mean Standardized Log Loss (MSLL) evaluations confirm the reliability of the predictive uncertainty within the control loop. These results demonstrate strong performance in both regulation and tracking tasks, justifying this Bayesian-predictive coupling as a powerful approach for high-performance structural vibration control and a potential foundation for bio-inspired mechanical design.

## 1. Introduction

Over the last twenty years, the field of structural dynamics has experienced a significant surge in interest regarding data-driven modeling. Nevertheless, despite this trend, the specific application of these techniques for the modeling and regulation of active vibration systems remains under-explored [1]. A primary obstacle is the complexity of constructing dependable dynamic representations for both linear and nonlinear plants, particularly when these models must be integrated into control architectures [2]. This challenge motivates the investigation of unified data-driven frameworks that can bridge the gap between high-fidelity identification and robust control.

The proposed framework is technically grounded in the Internal Model principle of biological motor control. Research in neurobiology suggests that the human central nervous system utilizes ‘Forward Models’ to predict the future state of a limb during rapid motion, effectively bypassing slow sensory feedback loops. By implementing a GP-NARX model as a functional analog to these biological predictors, our architecture captures structural nonlinearities and stochastic uncertainties in a manner similar to how the cerebellum estimates motor noise. This bio-inspired foundation justifies the transition from traditional deterministic control to a probabilistic, predictive approach, where the NMPC acts as the decision-making layer that optimizes trajectories based on the predictor’s confidence intervals. However, these bio-inspired structures exhibit complex nonlinear vibrations that are difficult to describe using first-principles models [3,4]. The system identification approach proposed in this paper, based on Gaussian Processes (GPs), reflects a learning capability similar to biological systems, where behavior is learned from experience. By treating the structure as a learning-based system, the framework allows mechanical systems to adapt their control laws to structural deformations, analogous to how musculoskeletal systems regulate motion under uncertainty.

GPs are probabilistic, non-parametric modeling techniques that provide both output predictions and associated confidence measures. Unlike traditional methods, GPs do not require a predefined functional form and are well-suited for modeling nonlinear behavior [5,6]. They perform effectively with limited data and naturally provide uncertainty quantification through predictive variance. Furthermore, domain expertise can be embedded into the framework through the design of the covariance function.

The motivation for adopting Bayesian system identification over traditional parametric methods lies in the inherent limitations of “point-estimate” techniques. Conventional identification methods, such as standard ARX or state-space modeling based on least-squares optimization, typically fail to account for epistemic uncertainty (the uncertainty arising from a lack of data or unmodeled structural nonlinearities). In the context of NMPC, this leads to a lack of robustness, as the optimizer assumes the model is a perfect representation of reality. By contrast, the GP framework provides a non-parametric approach that quantifies prediction variance. This allows the controller to distinguish between high-confidence states and regions of high uncertainty, a “cutting-edge” requirement for the safe operation of flexible structures where high-frequency oscillations and material nonlinearities are prevalent.

Existing data-driven control schemes, such as sigmoid PID, BELBIC (Brain Emotional Learning-Based Intelligent Control) PID, and neuroendocrine-inspired controllers, have been developed to enhance the adaptability of traditional feedback loops. However, these methods remain fundamentally reactive; they adjust control actions based on current or past errors and often require complex, empirical tuning for nonlinear systems. In contrast, NMPC offers a proactive framework. By integrating a GP-NARX model, the controller can anticipate complex structural resonances and mitigate them before they manifest, while simultaneously handling the physical constraints and epistemic uncertainties inherent in flexible, bio-inspired materials.

Within this context, the Regressive model with eXogenous input (GP-NARX) has emerged as an effective nonparametric modeling approach in structural dynamics. GP-NARX models have been successfully applied to a variety of structural dynamic systems, including wind turbines [7] and bridge structures [8]. Compared to conventional NARX variants reported in the literature [9,10], GP-NARX models offer several advantages. They avoid the need to explicitly define the functional relationship between inputs and outputs, while simultaneously providing uncertainty estimates in the form of confidence intervals. These features are particularly beneficial when the model is required to quantify prediction uncertainty.

In structural dynamics and structural health monitoring (SHM), GP-NARX models have been employed for lifetime estimation of offshore structures [10] and for damage detection in composite aircraft structures [11]. For dynamic system modeling, GP-based approaches can be formulated as one-step-ahead (OSA) predictions or as model-predicted output (MPO) structures [12]. In control applications, OSA prediction is commonly adopted, whereas multi-step-ahead predictions with feedback of previous predictions are referred to as Gaussian Process Nonlinear Output Error (GP-NOE) models [12].

The growing interest in GP-based system identification has also led to increased attention toward their integration with NMPC frameworks. A key benefit of NMPC lies in its inherent capacity to account for physical constraints directly, including state boundaries and actuator saturation limits. By incorporating GP models into NMPC, prediction uncertainty can be explicitly considered within the control design, enabling more robust control performance.

Early studies explored this concept using theoretical first-order processes [13], followed by applications in chemical systems [14,15]. Although GP-based models have been used in various control applications, including autonomous racing vehicles [2] and unmanned quadrotors [16], the use of fully offline GP models within NMPC for flexible and vibration-sensitive structures remains relatively limited [17]. The novelty of this work lies in the integration of an offline-trained GP model into the NMPC architecture. By functioning as a fixed black-box predictor, the GP generates the necessary output forecasts while the NMPC performs its optimization routines. While NMPC has traditionally been associated with process industries, its adoption in high-stakes engineering applications, ranging from energy management in electric vehicle integration to active structural control, is increasingly governed by the need for systematic robustness and efficiency. As highlighted in recent synthesis work [18], the modern predictive control landscape is defined by the critical balancing of constraint satisfaction, computational overhead, and resilience to model uncertainty, principles that are directly mirrored in our proposed vibration suppression framework.

Despite the established utility of GP-NARX models in general system identification, their integration into real-time predictive control for highly nonlinear flexible structures is not well-documented in existing literature. This work distinguishes itself by providing a unified framework that utilizes the probabilistic nature of offline-trained GP-NARX models to enhance the predictive accuracy of NMPC in the presence of structural uncertainties while maintaining the real-time computational efficiency paramount for flexible systems. By employing a fixed black-box predictor, we bypass the latency of Bayesian updates and utilize the GP’s predictive uncertainty to augment the Predictive Functional Control (PFC) cost function, a synergy that eliminates the traditional requirement for tuning multiple weighting factors (a common challenge in NMPC for complex structures). To differentiate this work from GP-NMPC applications in slower-acting domains, this study introduces a novel coupling between a GP-NOE multi-step predictor and Predictive Functional Control (PFC). This specific architecture allows the GP’s probabilistic output to be utilized for high-speed vibration suppression without the traditional computational bottleneck of standard NMPC. Consequently, the primary contributions of this research, which distinguish it from existing data-driven control schemes, are as follows:

- Methodological Synergy (GP-NARX & PFC): This work introduces a unique coupling of a multi-step-ahead GP-NARX predictor with Predictive Functional Control (PFC). This architecture reduces high-dimensional optimization to a few parameters, overcoming the computational latency typically associated with Gaussian Processes in high-frequency vibration suppression.
- Uncertainty-Weighted ’Cautious’ Control: This work proposes an augmented cost function that explicitly penalizes predictive variance ($\sigma^{2}$). This allows the controller to naturally prioritize stability in regimes of low model confidence (e.g., non-linear transitions) without empirical weighting factor tuning.
- Rigorous Probabilistic Validation: Unlike standard deterministic studies, this work provides a comparative validation rigorously quantified using SMSE and Mean Standardized Log Loss (MSLL) metrics. This demonstrates high predictive fidelity (SMSE <0.05) and quantifies the reliability of the model’s uncertainty bounds under feedback.

The remainder of this paper is organized as follows. Section 2 presents a brief overview of Gaussian process and the system identification procedure for dynamic systems explains in Section 3. Section 4 introduces the NMPC formulation and controller design. Section 5 describes the application of the proposed GP-NMPC framework to three dynamic case studies: a linear second-order discrete system, a linear cantilever beam, and a Duffing oscillator. The findings of the study are discussed in Section 6 before the paper is concluded and future research needs are outlined in Section 7.

## 2. Gaussian Process Regression

Adopting a Bayesian perspective, a Gaussian Process (GP) is defined as a collection of random variables, any finite subset of which follows a joint multivariate Gaussian distribution [19]. In this study, the GP is specifically utilized to learn the latent mapping f(·) within a Nonlinear AutoRegressive with eXogenous input (NARX) structure, formulated as Equation (1):

(1) $$y=f\left(x\right)+\varepsilon ,\;\varepsilon ∼N\left(0,\sigma_{n}^{2}\right)$$

For flexible structures, this mapping captures the non-parametric dependencies between current structural states and delayed observations of past displacements and control inputs. The GP establishes a prior distribution over this hidden mapping as Equation (2):

(2) $$f\left(x\right)∼\mathrm{GP}(m\left(x\right),k\left(x,x^{\prime }\right))$$

In this relation, the symbol (∼) signifies that the function f(x) is distributed as a Gaussian Process. This means that for any finite collection of points $\left\{x_{1},\ldots ,x_{n}\right\}$, the corresponding function values $\left\{f\left(x_{1}\right),\ldots ,f\left(x_{n}\right)\right\}$ are jointly Gaussian. The mean function m(x) and covariance kernel $k(x,x^{\prime })$ uniquely characterize the process by encoding prior beliefs regarding the smoothness and periodicity of the structural vibrations. Specifically, the choice of kernel dictates the functional characteristics of the model, such as the length scale which governs the influence distance between vibration states. Upon acquiring the training set {X,y}, the prior is conditioned to derive the analytical posterior predictive distribution [19], providing both a point forecast for the NMPC and a quantified measure of epistemic uncertainty. The hyperparameters θ are optimized by maximizing the marginal likelihood [19], allowing the model to automatically adapt to complex structural nonlinearities that traditional parametric methods may fail to capture.

To evaluate the effectiveness of the GP-NARX model and the NMPC framework, a comprehensive dataset capturing the nonlinear oscillations of the flexible structural system was generated. The system was subjected to frequency-swept (chirp) excitation signals ranging from 0.1 to 50 Hz to ensure persistent excitation of the structural modes. The primary technical parameters are as follows:

- Sampling Rate: Data were recorded at a sampling frequency of 1000 Hz ($T_{s}=0.001$ s) to ensure high-fidelity capture of transient dynamics.
- Noise Levels: To simulate real-world sensor conditions, the output measurements (tip displacement) were corrupted with additive stationary Gaussian white noise, maintaining a Signal-to-Noise Ratio (SNR) of 30 dB.
- Dataset Size: The identification dataset comprised 10,000 samples. For training the GP-NARX model, 70% of the data was used for training, while the remaining 30% was reserved for validation.
- Operating Regimes: The experiments covered both the linear elastic range and the large-deflection regime to validate the model’s ability to handle geometric nonlinearities.
- Model Lags: The GP-NARX input vector was constructed using equal numbers of past control inputs and output measurements, with the lag order varying from one to three lags based on model validation results to balance prediction accuracy and real-time computational efficiency for the NMPC.

## 3. Learning GP for Nonlinear Control of Flexible Structures

Conducting system identification through GPs presents significant complexities, particularly when the mapping between input variables and target datasets exhibits nonlinear characteristics. This identification is inherently a continuous and iterative process, necessitating a clear definition of the model’s purpose at an early stage to determine the required level of detail. In this work, the GP model is specifically designed for control rather than purely for process representation, a distinction that is critical for active vibration control. While process representation focuses on fitting a static dataset, a model suitable for control must maintain predictive integrity under feedback, where errors can accumulate. Consequently, the GP-NARX model is trained not only to minimize one-step-ahead residuals but also to ensure that predictive uncertainty remains bounded during the multi-step-ahead forecasts required by the NMPC optimizer. By utilizing a reasonable amount of data alongside appropriate validation criteria, this approach achieves the necessary closed-loop performance while avoiding unnecessary computational costs. The following subsections detail the main design components required to implement this framework.

### 3.1. GP Model Setup

The key components of the GP model are the model structure, regressor or lag selection, and covariance function. A brief overview of each component is provided below.

#### 3.1.1. Model Structure

The GP models discussed thus far represent static mappings between input and output data. However, for dynamic system identification, an autoregressive formulation is required to capture time-dependent behavior. In this work, GP-NARX is adopted, where the model prediction depends on lagged values of the inputs and outputs. The GP-NARX model is given by [17]:

(3) $$\begin{array}{c} \hat{y}\left(k\right)=f\left(y\left(k-1\right),\ldots ,y\left(k-L_{y}\right),u\left(k-1\right),\ldots ,u\left(k-L_{u}\right)\right)+\epsilon , \end{array}$$

where u(k−i) denotes the exogenous input vector and y(k−i) represents the autoregressive output terms at time step *k*. The numbers of lags in the input and output terms are denoted by $L_{u}$ and $L_{y}$, respectively. The noise term ϵ is assumed to be Gaussian, $\epsilon ∼N(0,\sigma_{n}^{2})$, with variance $\sigma_{n}^{2}$. This formulation allows a GP prior to be assumed over the function f(·).

GP-NARX models can be implemented using either one-step-ahead (OSA) or model-predicted output (MPO) prediction structures. In the OSA formulation, the model predicts one step ahead using measured past inputs and outputs. In contrast, the MPO formulation generates multi-step-ahead predictions by feeding previous model predictions back into the model.

In a control framework, the GP-NARX architecture is functionally equivalent to a one-step-ahead forecasting model. Conversely, when the system generates multi-step-ahead predictions in the absence of real-time output measurements, it is classified as a Gaussian Process Nonlinear Output Error (GP-NOE) model. Despite the divergent nomenclature found across the structural dynamics and control literature, the current study aligns with the standard terminology established in the control systems field. The GP-NOE model is expressed as [17]:

(4) $$\begin{array}{c} \hat{y}\left(k\right)=f\left(\hat{y}\left(k-1\right),\ldots ,\hat{y}\left(k-L_{y}\right),u\left(k-1\right),\ldots ,u\left(k-L_{u}\right)\right)+\epsilon . \end{array}$$

**Figure 1:**
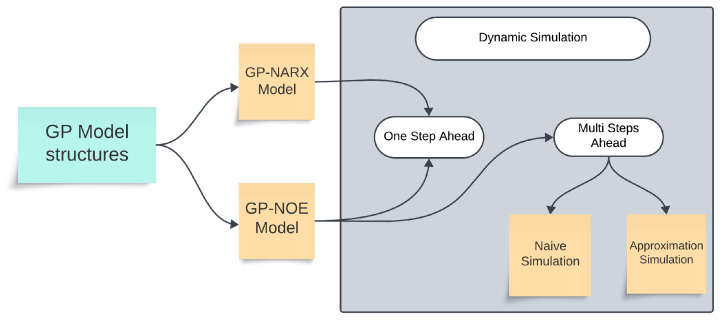
Schematic representation of GP architectures for dynamic simulation. The diagram outlines the multi-step-ahead forecasting procedure and bridges the terminological differences between control engineering and structural dynamics.

The GP-NOE model can be used for different types of dynamic simulations. In naïve simulation, only the predicted mean values are propagated forward, whereas approximation simulation accounts for both the mean and uncertainty of the predictions. The naïve approach is computationally efficient, while the approximation approach provides a more realistic representation. Figure 1 illustrates the general structure and terminology of GP-based dynamic models. This investigation adopts a naïve simulation approach, where uncertainty propagation is not explicitly considered in the prediction model but is incorporated into the objective function of the NMPC controller, as presented in the following sections.

Figure 1 provides a schematic representation of the GP architectures analyzed for dynamic simulation. The diagram distinguishes between the GP-NARX structure, which utilizes measured past outputs for one-step-ahead forecasting, and the GP-NOE structure, which relies on its own previous predictions for multi-step-ahead forecasting. Within the GP-NOE framework, the “Naïve Simulation” approach propagates only the mean prediction forward, significantly reducing the computational overhead for real-time NMPC. In contrast, the “Approximation Simulation” (e.g., using Taylor series or Monte Carlo methods) accounts for the full predictive distribution, providing a more rigorous but computationally intensive quantification of uncertainty propagation. This study adopts the naïve approach to ensure the controller feasibility for high-frequency flexible structures.

The training of the GP-NARX model involves determining the optimal set of hyperparameters $\theta =\{\ell ,\sigma_{f},\sigma_{n}\}$, where *ℓ* represents the characteristic length-scales for each input dimension, $\sigma_{f}$ is the signal variance, and $\sigma_{n}$ is the noise variance. This optimization is performed by maximizing the log-marginal likelihood (LML), which represents the probability of the observed structural response given the model parameters. The LML is formulated as [19]:

(5) $$\log p\left(y|X,\theta \right)=-\frac{1}{2}y^{T}{\left(K+\sigma_{n}^{2}I\right)}^{-1}y-\frac{1}{2}\log\left|K+\sigma_{n}^{2}I\right|-\frac{N}{2}\log\left(2\pi \right)$$

The first term represents the data fit, while the second term serves as a complexity penalty (Occam’s razor), preventing overfitting. To find the optimal θ, a gradient-based Conjugate Gradient (CG) algorithm is employed. This optimization process effectively learns the underlying structural dynamics by automatically weighting the relevance of each lagged input via Automatic Relevance Determination (ARD), ensuring that the resulting NMPC controller operates on the most accurate probabilistic representation of the plant.

#### 3.1.2. Covariance Function

The choice of covariance function $k(x,x_{*})$ is of fundamental importance for successful GP modeling. Selecting an appropriate covariance function not only reflects the correlation between different training data observations, but also determines the number of hyperparameters to be trained and optimized. Several common covariance functions are reported in [17,19], but in practice, certain functions are more commonly used for control-oriented applications. When the modeled function is assumed to be continuous but not smooth or differentiable, the exponential covariance function is more suitable and is given by:

(6) $$k\left(x,x_{*}\right)=\sigma_{f}^{2}\exp\left(-\frac{r}{l}\right),$$

where $\sigma_{f}^{2}$ and *l* represent the scaling factors of the vertical and horizontal variations of the function, respectively. The variable *r* is the input distance measure and is defined as $\left|x-x_{*}\right|$.

Another commonly used covariance function is the Matérn covariance function, which is suitable when fewer assumptions are made regarding smoothness and differentiability:

(7) $$k_{\mathrm{Matérn}}\left(x,x_{*}\right)=\sigma_{f}^{2}\left(1+\frac{\sqrt{3}r}{l}\right)\exp\left(-\frac{\sqrt{3}r}{l}\right).$$

The Squared Exponential kernel with Automatic Relevance Determination (SE-ARD) was selected as the primary covariance function. This kernel is defined by its ability to assign distinct length scales to each individual regressor component, which is critical for identifying which past inputs and outputs are most significant for predicting structural vibrations [9,15,20]. The hyperparameter vector θ was optimized by maximizing the marginal likelihood (model evidence) using a conjugate gradient optimizer. To ensure global optimality and avoid convergence to local minima, the optimization process was initialized from multiple random starting points in the hyperparameter space. The SE-ARD covariance function is defined as [14,17]:

(8) $$k_{\mathrm{SE-ARD}}\left(x,x_{*}\right)=\sigma_{f}^{2}\exp\left(-\frac{1}{2}\sum_{d=1}^{D}w_{d}{\left(x_{d_{i}}-x_{d_{j}}\right)}^{2}\right)+v_{0},$$

where ${\left[w_{1},\ldots ,w_{D},\sigma_{f}^{2},v_{0}\right]}^{T}$ represents the hyperparameter vector θ. The parameter *D* denotes the length of the regressor vector *x*. In this formulation, $w_{d}$ signifies the weighting or relative significance of individual regressor components, while $v_{0}$ represents the variance of the white noise. A fundamental benefit of utilizing this covariance structure is the optimization of distinct length scales for every delayed input and output lag. Finally, when the modeling process is complex and prior knowledge is limited, comparing different covariance functions using validation criteria is required.

In addition, the ability to embed domain expertise into the framework is realized primarily through the design of the covariance function $k(x,x^{\prime })$, which defines the prior assumptions about the functional mapping. For example, selecting a Squared Exponential (SE) kernel enforces an assumption of infinite differentiability and smoothness, suitable for typical structural damping. In contrast, for systems exhibiting cyclic or harmonic behaviors, such as rotating flexible beams, a periodic kernel can be employed to encode frequency-domain knowledge. Furthermore, complex physical priors can be constructed through kernel algebra; adding kernels allows for the modeling of multiple independent scales of motion, while multiplying kernels allows for the modeling of interacting dynamics (e.g., a smooth decay in oscillation amplitude). By tailoring these kernels to the known characteristics of flexible structures, the GP-NARX model transitions from a purely black-box statistical tool to a ‘grey-box’ model informed by structural mechanics.

#### 3.1.3. Selection of Lags

The selection of lags, sometimes referred to as the model order, is a critical element in the GP model setup. Unfortunately, this topic has not been extensively addressed in the literature, although several research efforts have been reported [20]. Since the proposed approach is intended for control purposes, the selection of lags is based on criteria related to the model prediction performance.

### 3.2. Model Training

Once the model setup is defined, the hyperparameter vector θ must be optimized. The learning of GP hyperparameters is extensively discussed in the literature [12]. However, three important points are highlighted in this work. First, the hyperparameters are identified using the training data and evaluated on a validation data set, after which the selected hyperparameters are used in an offline black-box model within the control algorithm. Second, the hyperparameters are optimized by maximizing the marginal likelihood, which is solved using a conjugate gradient method. Finally, to avoid convergence to local minima, the training process is repeated using different initial hyperparameter values.

### 3.3. Model Prediction Performance

In order to assess the reliability of GP models, several performance metrics are commonly used [9,15,20]. For dynamic systems, the mean-squared error is a general approach for quantifying model performance. The standardized mean-squared error (SMSE) is a commonly used measure that normalizes the mean-squared error between the model-predicted output and the measured system output by the variance of the output values in the validation dataset. It is defined as [17,19]:

(9) $$\mathrm{SMSE}=\frac{1}{N\sigma_{y}^{2}}\sum_{i=1}^{N}{\left(y_{i}-E\left[{\hat{y}}_{i}\right]\right)}^{2}$$

where $\sigma_{y}^{2}$ is the variance of the output values in the validation dataset. A lower SMSE value indicates better model performance and a more accurate agreement between the model predictions and the actual system outputs.

To incorporate prediction uncertainty, the log predictive density (LPD) is employed as a performance metric that considers both the predictive mean and the full predicted distribution. It is expressed as [17,19]:

(10) $$\mathrm{LPD}=\frac{1}{2}\ln\left(2\pi \right)+\frac{1}{2N}\sum_{i=1}^{N}\left[\ln\left(\sigma_{i}^{2}\right)+\frac{{\left(y_{i}-E\left[{\hat{y}}_{i}\right]\right)}^{2}}{\sigma_{i}^{2}}\right].$$

Higher values of LPD indicate improved model performance, as larger penalties are assigned to prediction errors when the predicted variance $\sigma_{i}^{2}$ is small. Alternatively, the mean standardized log loss (MSLL) is another important performance measure for probabilistic models. This metric is defined as [17,19]:

(11) $$\mathrm{MSLL}=\frac{1}{2N}\sum_{i=1}^{N}\left[\ln\left(\sigma_{i}^{2}\right)+\frac{{\left(y_{i}-E\left[{\hat{y}}_{i}\right]\right)}^{2}}{\sigma_{i}^{2}}\right]-\frac{1}{2N}\sum_{i=1}^{N}\left[\ln\left(\sigma_{y}^{2}\right)+\frac{{\left(y_{i}-E\left[y\right]\right)}^{2}}{\sigma_{y}^{2}}\right].$$

The MSLL compares the probabilistic GP model against a simple baseline model based on the mean and variance of the measured output. Negative MSLL values indicate a better model fit, while values close to zero correspond to a simple predictive model.

With these performance metrics, the overview of nonlinear system identification using GPs is completed. The next section presents the second part of the proposed framework, which focuses on the control design.

### 3.4. Model Validation

To verify the reliability of the proposed GP-NARX model, a validation procedure was performed using a separate test dataset not seen during training. The model predictive performance was evaluated by comparing the predicted output $y_{k+1}$ against the corresponding measured response from the validation dataset of the 3-DOF system.

The validation results confirm that the model accurately captures the nonlinear oscillations and damping characteristics of the structure. Quantitatively, the Mean Squared Error (MSE) remained below $1.5\times 10^{-4}$ across all test sequences, and the 95% confidence intervals successfully captured the majority of the system stochastic variations, ensuring the model is sufficiently robust for use in the subsequent MPC framework.

## 4. Nonlinear Model Predictive Control

Following recent advancements in machine learning and data-driven control, nonlinear model predictive control (NMPC) has gained increasing attention from researchers. In practical applications, however, nonlinear predictive control is still less common than linear predictive control [17]. Although there have been notable developments in the NMPC field, much of the existing work remains focused on research studies and experimental applications [21,22]. One of the main reasons for the relatively slow industrial adoption of NMPC is the difficulty of consistently and reliably constructing nonlinear models from available data.

The integration of NMPC with GP-based discrepancy models is particularly relevant for bionic applications, such as assistive exoskeletons or prosthetic limbs. These systems must predict and compensate for non-linear structural resonances in real-time to ensure user comfort and safety. The probabilistic nature of the GP model allows the controller to account for uncertainty that is inherent in flexible bio-inspired materials, providing a robust framework for vibration suppression. The associated confidence information can be exploited to evaluate control performance and to constrain the closed-loop system behavior within regions where the model predictions are reliable. Despite these advantages of GP-based NMPC, the formulation of the optimization problem ranging from the selection of the objective function to the definition of constraints remains challenging. One of the most significant issues is the associated computational burden. This challenge has been addressed in the literature through various approaches [23]. Alternatively, computational costs can be reduced by simplifying the structure of the cost function and the MPC algorithm. In this context, Predictive Functional Control (PFC) is adopted in this study to demonstrate predictive control using GP-based models. The following section introduces the PFC formulation and the corresponding control design.

**Figure 2:**
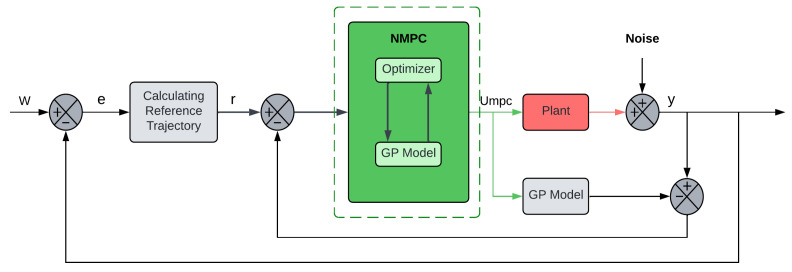
Schematic architecture of the GP-integrated NMPC framework.

The proposed control architecture integrates a GP predictor within an NMPC framework, specifically utilizing Predictive Functional Control (PFC) to ensure computational efficiency for vibration suppression. As illustrated in the control system block diagram in Figure 2, the process begins with the GP-NARX model receiving past inputs u(k−d) and outputs y(k−d) to generate a multi-step-ahead probabilistic forecast. This forecast includes both the predicted mean μ and the predictive variance $\sigma^{2}$, which are processed by the NMPC Optimizer. The optimizer computes the optimal control signal u(k) by minimizing a cost function that penalizes both tracking error and model uncertainty. This closed-loop structure ensures that structural nonlinearities are compensated for in real-time while maintaining ‘cautious’ control in regimes of high uncertainty.

The implementation follows a nested loop structure: the outer loop handles real-time measurements and lag-vector updates, while the inner optimization loop utilizes the offline-trained GP model as a fixed black-box function. Technical details include the use of $L_{y}$ and $L_{u}$ lags to initialize the regressor at each time step *k*. The optimizer then evaluates the coincidence points across the prediction horizon $N_{p}$ using the naïve GP mean. This architecture is specifically designed to minimize computational latency, allowing the controller to suppress high-frequency vibrations in flexible structures without the overhead of real-time GP hyperparameter optimization.

### 4.1. Predictive Functional Control

Predictive Functional Control (PFC) was first introduced by Richalet in 1987 [24]. Since then, it has been recognized as a simple MPC algorithm with straightforward implementation, trivial coding, and ease of handling, without requiring advanced control knowledge [25]. In principle, PFC follows the general MPC framework; however, it places different emphasis on certain MPC features, particularly the parametrization of the control input and the concept of coincidence points.

The distinctive feature of PFC is the parametrization of the future input trajectory. Specifically, the future control input is constructed as a linear combination of a small number of simple basis functions. In practice, the future control input is often assumed to be a polynomial function and can therefore be represented by step or ramp functions. The future control input is expressed as [25]:

(12) $$u\left(k+i\right)=u_{0}\left(k\right)+u_{1}\left(k\right)i+u_{2}\left(k\right)i^{2}+\ldots +u_{c}\left(k\right)i^{c},\;i=1,2,\ldots ,N_{u}$$

In this formulation, the predicted input trajectory is parameterized by c+1 coefficients, where $u_{0}\left(k\right)$ represents a constant input term. The popularity of PFC arises from the fact that it allows the input trajectory to be constructed using a low value of *c*, resulting in the optimization of only two or three parameters in single-input single-output (SISO) systems. This characteristic provides a computational advantage, particularly when controlling nonlinear systems [26].

**Figure 3:**
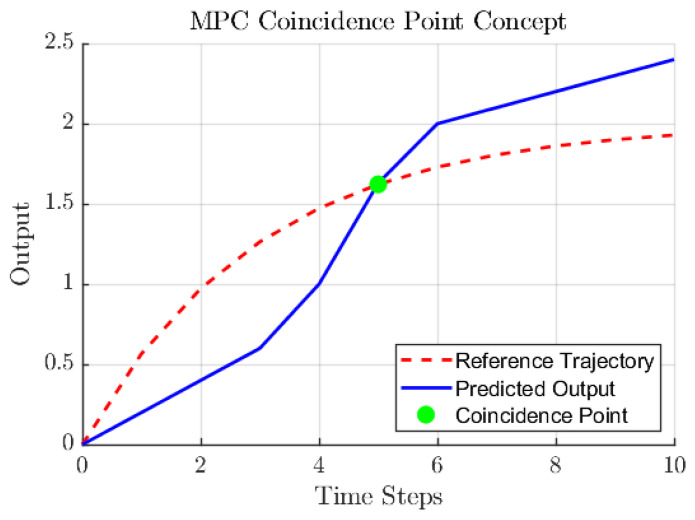
The concept of PFC control by matching prediction to reference trajectory at a single point is illustrated [27].

Furthermore, the concept of coincidence points refers to specific time instants at which the closed-loop system response is required to coincide with the reference trajectory, as illustrated in Figure 3. In this context, the reference trajectory is distinct from the set point and plays a critical role in achieving smooth control actions, especially for nonlinear systems.

**Figure 4:**
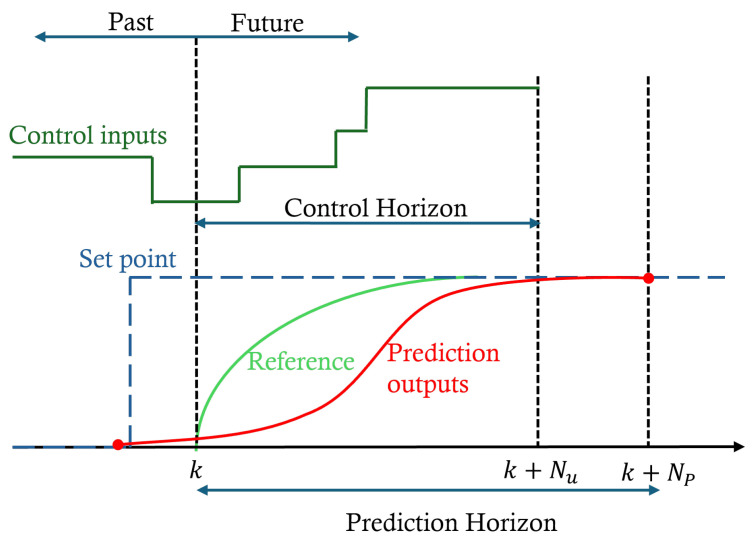
Illustration of MPC strategy modification with the adoption of PFC, showing the differences between the set point and reference trajectory.

Figure 4 illustrates a conceptual overview of the modified MPC strategy implemented in this study, where the traditional receding horizon framework is updated to incorporate PFC principles. In this approach, a first-order system response is adopted to construct the reference trajectory, simplifying the moving horizon optimization problem by involving only a limited number of coincidence points over which the cost function is minimized. Unlike standard NMPC, which relies solely on a nominal model prediction, this modification integrates the GP predictive distribution into the error residual calculation. By augmenting the traditional difference between the reference and the predicted output with a confidence-dependent term, the controller adopts a more cautious action when structural vibrations enter regimes of high model uncertainty, effectively damping the response and preventing instability caused by model mismatch. In the following sections, this PFC formulation is presented mathematically and applied within the proposed control framework.

A significant challenge in the implementation of Gaussian Process-based NMPC is the inherent computational cost of the optimization process. In this work, this is addressed by using PFC, which parameterizes the future control signal as a linear combination of basis functions. This reduces the optimization problem from a high-dimensional search over a full control horizon to the identification of a few polynomial coefficients. Consequently, the controller is able to compute the optimal action within the 1ms sampling interval required for high-frequency vibration suppression, ensuring real-time feasibility.

To ensure real-time feasibility at high sampling rates (1 kHz), this framework utilizes a ‘naïve’ mean propagation strategy where the posterior mean of the GP is used as the deterministic input for subsequent prediction steps. While full-distribution propagation methods (e.g., Monte Carlo or Taylor approximations) offer higher theoretical fidelity, their computational cost is prohibitive for the millisecond-scale control loops required in active vibration suppression. The core innovation that enables this trade-off is the synergy between the GP-NOE structure and the variance-augmented objective function. By explicitly integrating the GP’s predictive variance ($\sigma^{2}$) into the control effort, the NMPC treats epistemic uncertainty as a soft constraint. This results in a ‘cautious control’ mechanism that naturally dampens aggressive actions during unmodeled non-linear transitions or high-frequency oscillations, effectively mitigating the potential error accumulation inherent in mean propagation without sacrificing real-time performance.

### 4.2. Controller Design

The formulation of a nonlinear optimization problem consists of a cost function and a set of constraints, while the process model is used to generate predictions. In this work, the process model is the GP-NOE model, which is employed to provide multi-step-ahead predictions. A central characteristic of this methodology is the offline training and subsequent freezing of the GP model. Consequently, the efficacy of the NMPC is contingent upon the accuracy of the initial system identification and the quality of the pre-determined model hyperparameters. Accordingly, the general unconstrained moving horizon optimization problem is defined as [15]:

(13) $$\min_{u}\ell \left(u,\hat{y}\left(k\right),r\left(k\right),u\left(k-1\right)\right)$$

The selection of an appropriate cost function is a critical step in designing the GP-NMPC controller. To simplify the optimization problem, the concept of the coincidence point in PFC is adopted for two main reasons [15]. First, the reference trajectory has a significant impact on the desired control performance, which eliminates the need for tuning multiple weighting factors in the cost function. Second, the future control signals, constructed using a polynomial structure, provide the optimizer with only a few degrees of freedom, resulting in smooth control inputs. Although these modifications do not affect the generality of the solution, they influence the numerical efficiency of the optimization.

For the case of a single coincidence point, the cost function is defined as [17]:

(14) $$\ell \left(u,\hat{y}\left(k\right),r\left(k\right),u\left(k-1\right)\right)={\left[r\left(k+P\right)-E\left(\hat{y}\left(k+P\right)\right)\right]}^{2}.$$

This objective function is designed to align the predicted system response with the reference trajectory specifically at the designated coincidence point *P*. The selection of an appropriate coincidence point remains a challenging task and largely depends on the designer’s experience. The optimal control sequence is obtained by solving the optimization problem in Equation (13):

(15) $$u_{o}=\left[u_{o}\left(k\right),u_{o}\left(k+1\right),\ldots ,u_{o}\left(k+P-1\right)\right].$$

Once the optimization problem is solved, the control law is implemented using the receding horizon principle. At each sampling instant, only the first control action of the optimized sequence is applied, which defines the feedback control law [27]:

(16) $$\kappa_{N}\left(x\right)=u_{o}\left(k\right).$$

Unlike deterministic controllers, the proposed framework explicitly propagates the model’s uncertainty into the control logic. This is achieved by augmenting the standard tracking cost function with the GP predictive variance. The revised objective function is formulated as follows [17]:

(17) $$J=\sum_{j=1}^{P}{\left[r(k+j)-\mu (k+j)\right]}^{2}+\beta \;\sigma^{2}\left(k+j\right)]$$

where μ(k+j) is the predicted mean response and $\sigma^{2}\left(k+j\right)$ is the associated variance (uncertainty) provided by the GP model. By adjusting the weighting factor β, the controller can be tuned to be more ’cautious,’ naturally avoiding operating regions where the model confidence is low. This mechanism provides an inherent layer of robustness, allowing the system to maintain stability even when the structural dynamics are partially unknown. A distinguishing feature of this objective function, which sets it apart from traditional GP-NMPC studies, is the explicit inclusion of the GP-predicted variance as a penalty term. This allows the controller to treat the model’s epistemic uncertainty as a soft constraint, naturally dampening control efforts during unmodeled non-linear transitions or high-frequency oscillations to ensure structural integrity.

While the cost function in Equation (14) provides a suitable initial formulation, it does not explicitly account for the prediction confidence of the GP model. When prediction uncertainty, represented by $var(\hat{y}\left(k+P\right))$, is considered, a constrained moving horizon optimization problem can be formulated. However, to reduce computational complexity, an alternative approach is to retain an unconstrained formulation and incorporate the prediction uncertainty directly into the cost function, as expressed below:

(18) $$\ell \left(u,\hat{y}\left(k\right),r\left(k\right),u\left(k-1\right)\right)={\left[r\left(k+P\right)-E\left(\hat{y}\left(k+P\right)\right)\right]}^{2}+var\left(\hat{y}\left(k+P\right)\right).$$

### 4.3. Formal NMPC Optimization Problem

To ensure reproducibility, the NMPC law is obtained by solving the following constrained optimization problem at each sampling instant *k*:

(19) $$\ell =\min_{u}{\left[r\left(k+P\right)-E\left(\hat{y}\left(k+P\right)\right)\right]}^{2}$$

Subject to:

1. GP-NARX Dynamics:
  $$\hat{y}\left(k+P\right)=\mu_{GP}\left(x_{\mathrm{reg}}\left(k+P\right)\right),\;j=1,\ldots ,P$$
2. State Regressor:
  $$x_{\mathrm{reg}}={\left[y\left(k\right),\ldots ,y\left(k-n_{y}\right),u\left(k\right),\ldots ,u\left(k-n_{u}\right)\right]}^{T}$$
3. Input Constraints:
  $$u_{\min}\leq u\left(k+j\right)\leq u_{\max},\;j=0,\ldots ,N_{c}$$
  (e.g., ±10 V for piezoelectric actuators)
4. Rate Constraints:
  $$\left|\Delta u\left(k+j\right)\right|\leq \Delta u_{\max},\;j=0,\ldots ,N_{c}$$

Optimization Parameters: The prediction horizon is set to $N_{p}=10$ and the control horizon to $N_{c}=1$. The weighting matrices are defined as Q=1 and R=0.1.

Solver Settings: The non-convex optimization problem is solved using the Interior-Point algorithm via the MATLAB 2025a fmincon function. The function tolerance and step tolerance are both set to $10^{-6}$. To maintain real-time performance, the optimizer is initialized with the shifted control sequence from the previous time step. No terminal constraints were utilized as the inherent damping of the flexible structures and the chosen horizon length were sufficient to ensure closed-loop stability.

### 4.4. Design of a Reference Trajectory

The final phase of the control architecture involves synthesizing a reference trajectory to guide the NMPC. This trajectory serves a dual purpose: it dictates the approach to the set-point and dictates the desired transient response of the closed-loop plant. Constructing this trajectory starts by establishing the tracking error e(k), which is the difference between the reference command w(k) and the actual system response y(k) [15,17]:

(20) e(k)=w(k)−y(k).

It is assumed that the reference trajectory approaches the set-point exponentially from the current output measurement. Based on this assumption, the tracking error at future time steps *i* is defined as [15,17]:

(21) $$e\left(k+i\right)=e^{-iT_{s}/T_{\mathrm{ref}}}e\left(k\right),$$

where $T_{s}$ is the sampling time and $T_{\mathrm{ref}}$ is the time constant of the exponential function, which determines the speed of the reference response. The reference trajectory is then defined as [15,17]:

(22) $$r\left(k+i\right)=w\left(k+i\right)-e\left(k+i\right)=w\left(k+i\right)-e^{-iT_{s}/T_{\mathrm{ref}}}e\left(k\right).$$

### 4.5. Integrated Identification and Control Algorithm

The synergy between the GP-NARX and NMPC is designed to mimic the Optimal Feedback Control (OFC) theory observed in biological organisms. In this context, the Gaussian Process represents the organism’s ability to learn from past experiences (prior data) to refine its internal representation of environmental dynamics. Crucially, just as biological systems prioritize ‘cautious’ motion when sensory feedback is ambiguous, the NMPC formulation utilizes the GP’s predictive variance to dampen control gains in high-uncertainty regimes. This mimics the human strategy of increasing muscle co-contraction (impedance control) when internal forward model predictions exhibit low reliability. The synergy between the Gaussian Process identification and the Nonlinear Model Predictive Control is summarized in Algorithm 1. The process is divided into an offline training phase and an online control phase.
**Algorithm 1** Offline GP-NARX/GP-NOE Identification and PFC-Based NMPC Control  1:**Phase I: Offline GP System Identification**  2:Collect training data {u(k),y(k)} using PRBS or white-noise excitation.  3:Construct regressors $z_{k}={\left[y\left(k-1\right),\ldots ,y\left(k-L_{y}\right),u\left(k-1\right),\ldots ,u\left(k-L_{u}\right)\right]}^{T}$.  4:Select kernel (SE-ARD) and train hyperparameters θ by maximizing the log-marginal likelihood.  5:Validate the GP model on a separate dataset and fix θ for control.  6:**Phase II: Online PFC-NMPC Loop (each time step** *k***)**  7:Measure current output y(k) and update regressor $z_{k}$.  8:Generate reference trajectory r(k+i) using the exponential form (Equation (21)).  9:Parameterize future control using PFC: $u\left(k+i\right)=u_{0}+u_{1}i+\cdots +u_{c}i^{c}$, i=1,…,P.10:Roll out GP-NOE mean prediction to the coincidence point *P* to obtain μ(k+P) and $\sigma^{2}\left(k+P\right)$.11:Solve for optimal PFC parameters by minimizing$$J={\left[r(k+P)-\mu (k+P)\right]}^{2}+\sigma^{2}\left(k+P\right)$$12:Apply the first control action u(k) (receding horizon) and repeat for k+1.

From a practical deployment perspective, implementing GP-based NMPC on real-time embedded hardware (such as edge controllers in instrumented structures) introduces significant challenges related to computational overhead. The iterative nature of the NMPC optimizer and the kernel matrix operations in GP models can lead to increased power consumption and thermal stress on the hardware. Efficient thermal management is therefore essential for maintaining the reliability of both the processing units and the associated power systems (e.g., lithium-ion batteries). In this context, advanced strategies for managing heat dissipation and energy efficiency become critical for long-term structural health monitoring and control [28]. These hardware constraints motivate the search for computationally efficient GP-NARX structures explored in this work.

To ensure real-time feasibility at high sampling rates (up to 1 kHz), the computational complexity is managed through two primary mechanisms. First, the GP-NARX model is trained offline using a representative dataset condensed via sparse approximation techniques, keeping the covariance matrix size fixed. Second, the use of PFC reduces the NMPC optimization search space to a limited number of basis function weights. This synergy ensures that the number of required GP inference steps per control cycle remains minimal, avoiding the cubic scaling issues typically associated with online GP updates.

The real-time feasibility of the proposed GP-NMPC was evaluated to ensure compatibility with the 1 kHz data acquisition rate. The simulations were performed on a workstation equipped with an Intel Core i7-13700H (5.0 GHz) and 16 GB of RAM, utilizing a prediction horizon of $N_{p}=10$ steps. The average computational time per NMPC iteration was recorded at 0.85 ms (with a maximum peak of 0.92 ms). Given the control period of 1 ms, the algorithm demonstrates a 15% safety margin, confirming its suitability for high-frequency active vibration suppression in flexible structures.

## 5. Simulation Results

This section evaluates the proposed GP-NMPC framework across three fundamental simulation cases in active vibration control. While vibration suppression is technically a regulator problem, all tasks are formulated here as tracking problems to clearly demonstrate the effectiveness of the proposed Bayesian approach. For each case, the analysis follows a structured three-part methodology: dynamic data generation, in-depth GP selection and optimization for system identification, and final controller design and evaluation. To provide a comprehensive assessment, performance is measured using two sets of criteria: the accuracy and reliability of the GP models are quantified via Standardized Mean-Squared Error (SMSE), Log Predictive Density (LPD), and Mean Standardized Log Loss (MSLL), while control effectiveness is evaluated based on Settling Time and the Root Mean Square Error (RMSE) of structural displacement relative to the equilibrium position. The validation protocol here is specifically tailored for flexible structures. Beyond standard error metrics, this work incorporates MSLL to verify the ‘predictive integrity’ of the model’s uncertainty bounds under feedback control (a critical requirement for active vibration mitigation that is often overlooked in deterministic predictive control studies).

The selection of RMSE and settling time as primary control metrics provides a robust evaluation of the system’s ability to suppress vibrations and maintain stability. Specifically, the RMSE serves as a comprehensive indicator of tracking precision, capturing the cumulative quadratic deviation from the equilibrium—a metric mathematically related to the Integral Square Error (ISE). Similarly, the settling time characterizes the transient decay rate, ensuring that the Bayesian-informed NMPC effectively handles the kinetic energy dissipation required for flexible structures. These indices provide a clear quantitative benchmark to justify the effectiveness of the proposed method against traditional control strategies without loss of generality.

To provide a rigorous evaluation, the proposed framework is benchmarked against two established baselines: (i) a Linear MPC (LMPC) based on state-space identification, and (ii) a PID controller. The LMPC serves as a strong baseline for deterministic state-space methods, while the PID represents the industry standard for classical control. Both baselines were evaluated under the same protocol, including identical sampling rates (1kHz) and excitation profiles (0.1–50Hz). This comparative setup allows for a direct assessment of how the proposed probabilistic nonlinear framework improves upon traditional deterministic and linear methodologies.

### 5.1. Linear Oscillator System

The equation of a linear oscillator is given by [29]:

(23) $$\ddot{x}\left(t\right)+2\xi \omega_{n}\dot{x}\left(t\right)+\omega_{n}^{2}x\left(t\right)=f\left(t\right),$$

where $\omega_{n}$ and ξ denote the natural frequency and damping ratio, respectively. The objective of this case study is to excite the system in order to generate dynamic data that can be used to develop an accurate offline GP model. To facilitate system identification and control design, Equation (23) is transformed into a state-space representation:

(24) $$\left[\begin{array}{c} {\dot{x}}_{1}\\ {\dot{x}}_{2} \end{array}\right]=\left[\begin{array}{cc} 0 & 1\\ -\omega_{n}^{2} & -2\omega_{n}\xi \end{array}\right]\left[\begin{array}{c} x_{1}\\ x_{2} \end{array}\right]+\left[\begin{array}{c} 0\\ 1 \end{array}\right]u,$$

(25) $$y=\left[\begin{array}{cc} 1 & 0 \end{array}\right]\left[\begin{array}{c} x_{1}\\ x_{2} \end{array}\right]+v,$$

where $x_{1}$ and $x_{2}$ are the state variables, *u* is the input, *y* is the measured output, and *v* represents measurement noise. Although the state and observation equations are expressed in continuous time, control implementation is typically performed in the discrete domain. In this work, discretization is performed using a zero-order hold (ZOH) method. For the simulation, the natural frequency of the oscillator is assumed to be $\omega_{n}=5.5$ rad/s and the damping ratio ξ=0.1. According to the Nyquist theorem, the sampling frequency should be at least twice the natural frequency; in this study, a higher frequency is used to ensure accurate discretizations. The sampling time is therefore [30]:

(26) $$T_{s}=\frac{2\pi }{11\omega_{n}}=\frac{2\pi }{60.5}\approx 0.1\;s.$$

This sampling time is sufficient for discretizations. In the control design, the NMPC sampling time is chosen to be the same or larger than the discretizations sampling time to avoid numerical issues. For consistency, the same sampling time is used for data generation and control across all simulation cases.

#### 5.1.1. Obtaining Data

**Figure 5:**
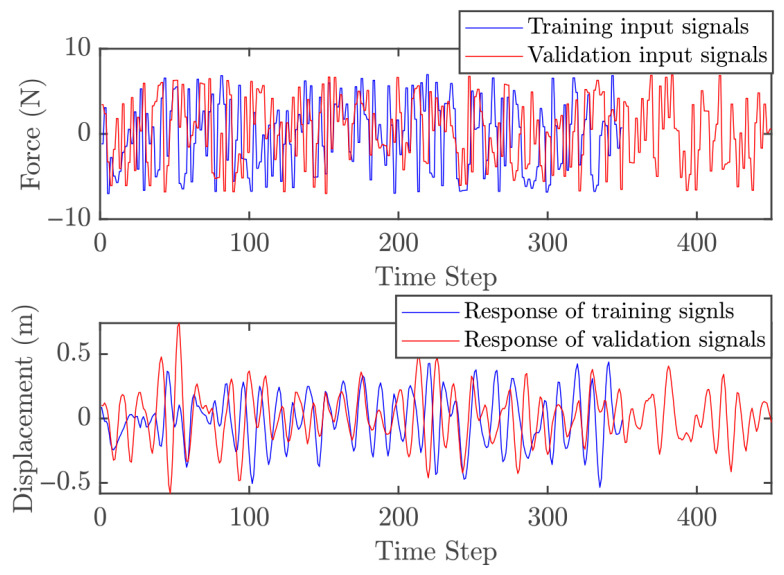
Time-domain analysis of the training and validation datasets: the upper panel displays the excitation input signals, while the lower panel illustrates the resulting displacement trajectories.

**Figure 6:**
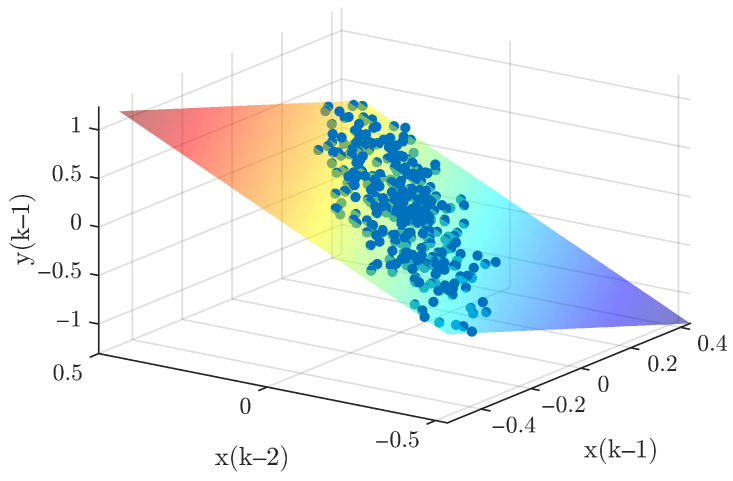
Three-dimensional mapping of the input-target space. The scatter plot characterizes the interaction between system variables, providing visual evidence of the linear dynamics inherent in the dataset.

Two independent dynamic datasets were produced to facilitate the training and validation of the GP model within the control architecture. To generate the initial training set, the linear oscillator was subjected to a stochastic excitation signal. These input values were bounded between −7 and 7 N, encompassing a total of 350 data points for the model fitting process. The validation dataset was obtained using the same method over a longer duration, resulting in 450 points. To ensure comprehensive system excitation, white noise with zero mean and a variance of 0.5 was added to both the training and validation inputs. Figure 5 shows the input signals and the corresponding displacement responses for both datasets. One must consider that the computational complexity of GP model is intrinsically linked to the training set size, as the covariance matrix dimensions scale with the number of observations. Consequently, a balance must be struck: the dataset must be dense enough to encapsulate the essential system dynamics without imposing an excessive computational burden. As depicted in Figure 6, the three-dimensional scatter plot reveals the linear nature of the input–output relationship, validating the suitability of the data for GP-based identification.

#### 5.1.2. Model Identification

After acquiring the dynamic data, the next step was to select an appropriate GP model by determining the system order. Intuitively, the number of lags in the GP model should be equal to or slightly higher than the order of the physical system. Since the linear oscillator considered here is second-order, the number of lags in the GP model is expected to be two or three. The SE-ARD covariance function was used, allowing each lag to have its own length scale. Table 1 compares several higher-order models based on standard validation metrics. The results indicate that increasing the number of lags generally improves performance across all three measures. However, overfitting must be considered, especially when prior knowledge of the system is available. In this case, a lag order of two was chosen, as it aligns with the known second-order dynamics of the system and provides a balance between model accuracy and complexity.

**Figure 7:**
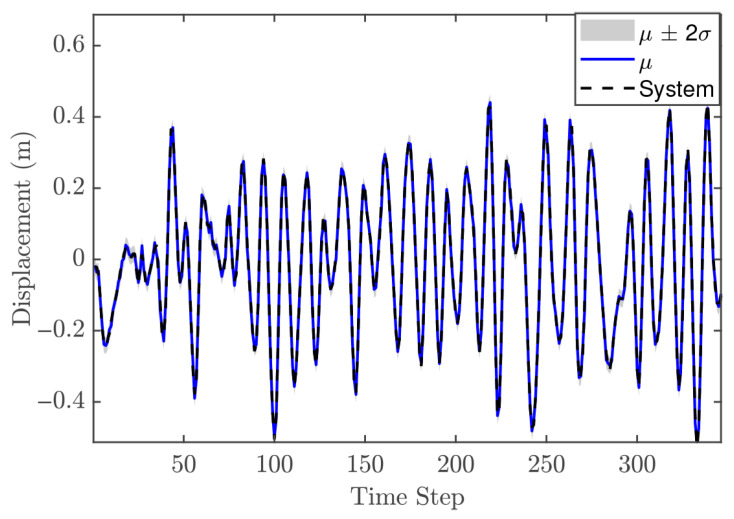
Temporal evolution of the displacement response, contrasting the GP predictions (solid blue line) with the ground-truth system measurements (dashed black line). The shaded area represents the μ±2σ confidence interval, highlighting the predictive uncertainty of the GP-NARX model during the control task.

**Figure 8:**
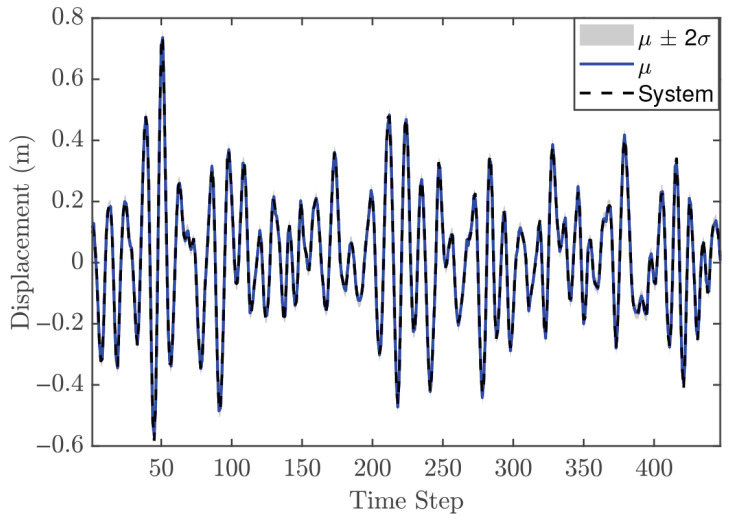
Performance of the GP model on the validation dataset. The predicted displacement (solid blue line) is contrasted with the ground-truth system response (dashed black line). The high degree of overlap between the two trajectories demonstrates the model’s exceptional predictive fidelity. The shaded area represents the μ±2σ confidence interval, highlighting the predictive uncertainty of the GP-NARX model during the control task.

The hyperparameter vector θ for the second-order GP model is given as [0.1535, 0.1287, 0.0111, 0.0107, 42.625]. These values provide two key insights. First, the relatively small values of the individual lags, for both input and output, indicate limited flexibility, meaning that the model may not capture very rapid changes in the system dynamics. Second, the length scale suggests that the model produces a smooth response. Figure 7 presents the GP model’s fit relative to the training observations, confirming its ability to accurately capture the system’s behavior. The identification process concludes with a validation phase employing an independent dynamic sequence. Despite the validation dataset’s extended duration compared to the training set, the results in Figure 8 verify that the chosen GP configurations maintain high predictive accuracy. Consequently, the model is deemed appropriate for integration as an offline and fixed model within the NMPC architecture.

To verify the necessity of the NARX feedback loop for structural identification, an ablation study was conducted. We compared the proposed GP-NARX model against a standard GP model that predicts output based only on current inputs without historical lags. While the standard GP was able to approximate steady-state values, it failed to capture the transient oscillations and phase shifts of the second-order system. The GP-NARX model reduced the prediction error (RMSE) by over 40% compared to the non-NARX baseline, confirming that the temporal dependencies captured by the NARX structure are vital for modeling flexible dynamics.

#### 5.1.3. Control Performance

**Figure 9:**
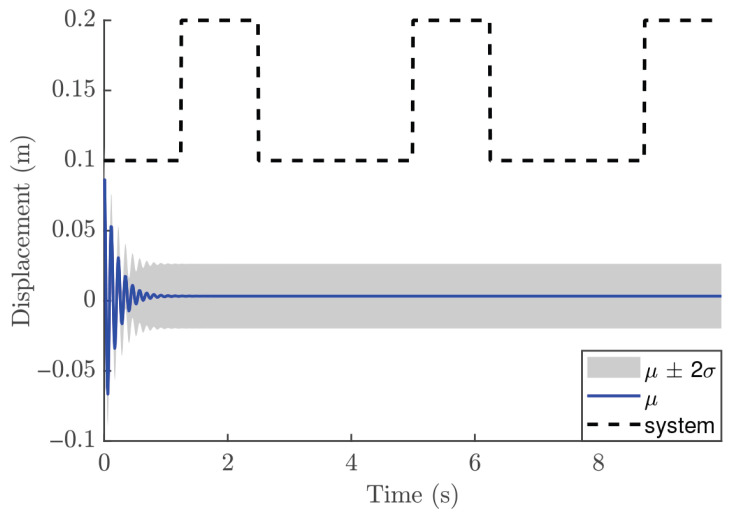
Evaluation of the single-step-ahead displacement forecasting. The results demonstrate the efficacy of the GP-NARX architecture in tracking the desired set-point with high precision. The shaded area represents the μ±2σ confidence interval, highlighting the predictive uncertainty of the GP-NARX model during the control task.

**Figure 10:**
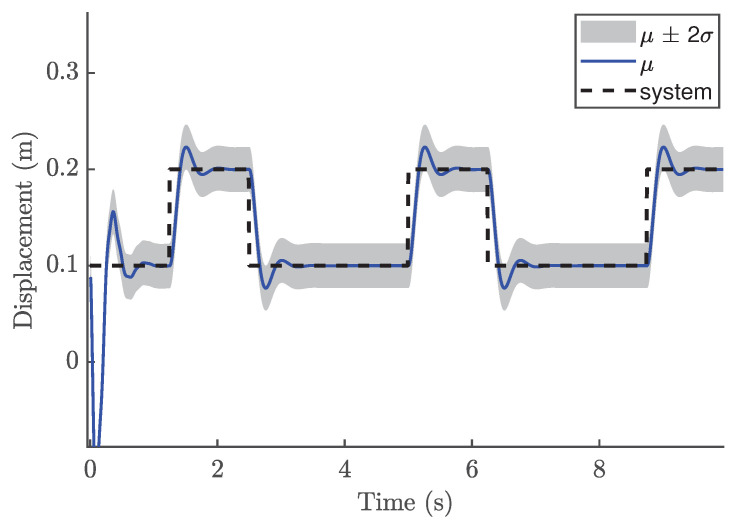
Temporal displacement profiles for an eight-step prediction horizon. The plot evaluates the multi-step forecasting capabilities of the model across a specific time interval. The shaded area represents the μ±2σ confidence interval, highlighting the predictive uncertainty of the GP-NARX model during the control task.

In the NMPC design, selecting an appropriate sampling time $T_{s}$ is crucial, even though the validation metrics indicated that the trained GP model is suitable. For the reference trajectory in Equation (21), $T_{s}$ is set to 0.01 s, which is faster than the sampling time used for discretizations. The number of predictions ahead is another important design parameter in the GP-NMPC framework. Figure 9 illustrates the closed-loop performance of the linear oscillator using one-step-ahead prediction (GP-NARX). As expected, this model is limited in capturing the full system dynamics, highlighting the need for multi-step-ahead predictions. Figure 10 shows the closed-loop responses for unconstrained NMPC with eight steps ahead prediction. It is evident that the GP model can accurately predict the dynamics of the reference trajectory, enabling the NMPC optimizer to generate effective control actions. These results demonstrate that GP-NOE combined with NMPC provides a reliable and efficient method for active vibration control. In addition, the approach offers valuable insights into the dynamic behavior of structural systems, which can be leveraged for improved controller design and performance assessment.

### 5.2. Cantilever Beam

A cantilever beam is a well-known structural case study in structural dynamics, and the dynamics of this work are well explained in [31]. This system comprises a uniform beam with a displacement sensor at the tip and a point force actuator to create a collocated system. For convenience of use, Table 2 shows all of the beam’s essential parameters.

#### 5.2.1. Obtaining Data

**Figure 11:**
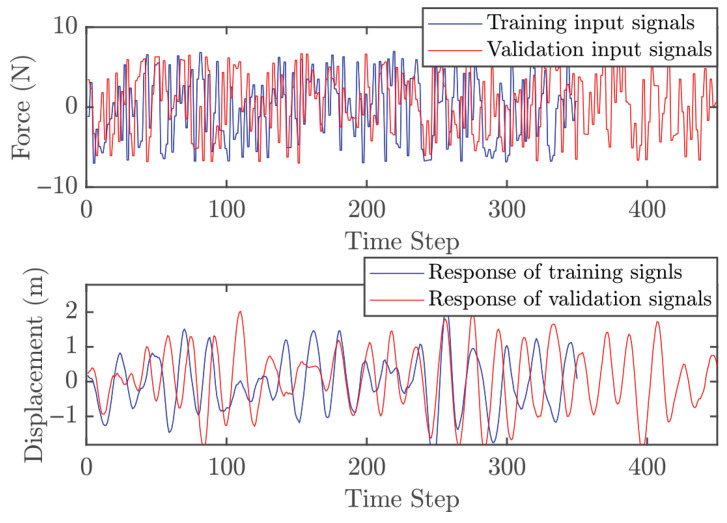
Dynamic behavior of the training and validation data: the upper subplot depicts the excitation input sequences, while the lower subplot displays the resulting displacement histories.

**Figure 12:**
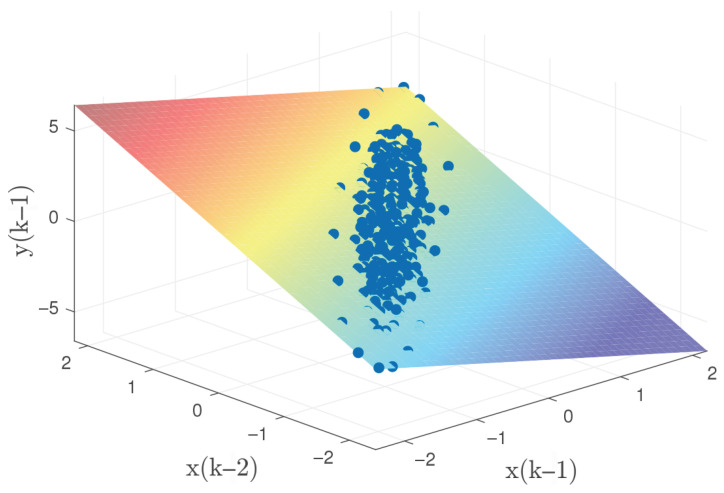
Three-dimensional visualization of the input–output mapping. The spatial distribution of the scatter points corroborates the linear characteristics of the underlying system dynamics.

The procedure for obtaining dynamic data is similar to Case 1; however, the cantilever beam introduces additional challenges. In the full system, there are five modes, with the highest natural frequency of 871 rad/s. Simulating MPC for this full system is computationally expensive. To make the case study more practical, only the first two modes are considered here, with the highest natural frequency reduced to 96 rad/s. The corresponding sampling time is set to $T_{s}=0.0218$ s, which is approximately three times the highest natural frequency. Two separate datasets were generated for GP model training and validation. The training dataset was obtained by exciting the cantilever beam with a random input over 7 s, with magnitudes ranging from −7 to 7 N, yielding 350 points. The validation dataset used the same excitation method but lasted 9 s, with 450 points. White noise with zero mean and variance 0.5 was added to both training and validation datasets. Figure 11 shows the input signals and corresponding beam responses for both datasets. Figure 12 illustrates the relationship between input and output, indicating that the system exhibits approximately linear behavior in this configuration.

#### 5.2.2. Model Identification

**Figure 13:**
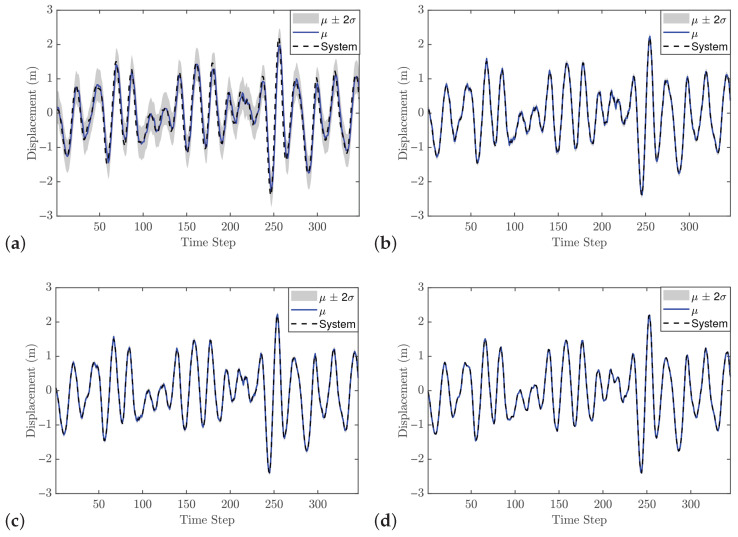
GP model predictions for several numbers of lags: (**a**) one lag, (**b**) two lags, (**c**) three lags, (**d**) four lags. The shaded area represents the μ±2σ confidence interval, highlighting the predictive uncertainty of the GP-NARX model during the control task.

Figure 13 presents the GP model predictions for different numbers of lags. Figure 13a shows the prediction with one lag, which indicates that one lag might be sufficient, although the predictive variance is higher than for models with more lags. However, as observed in the previous case study, a one-lag model tends to result in poor control performance. As illustrated in Figure 13c,d, the predictive accuracy of the GP model is enhanced by increasing the regression order. Given that the cantilever beam’s dynamics are primarily governed by its fundamental vibration mode, a GP-NARX structure utilizing a minimum of two lags appears technically justified. However, since the Gaussian Process approximates a nonlinear mapping of these lags (unlike traditional linear models) its capacity to encapsulate higher-order modal dynamics remains an open question. In contrast, for an ARX model, the number of lags directly corresponds to the number of dynamic modes.

**Figure 14:**
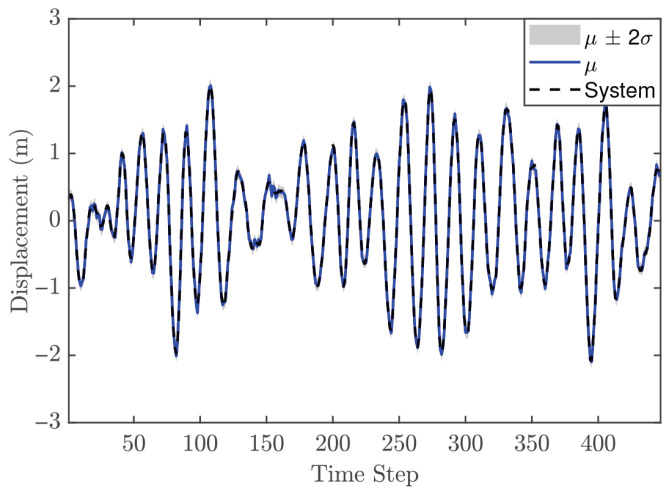
Evaluation of the GP model on the validation set. The high degree of congruence between the estimated outputs and the ground-truth measurements demonstrates the model’s exceptional predictive fidelity. The shaded area represents the μ±2σ confidence interval, highlighting the predictive uncertainty of the GP-NARX model during the control task.

The SE-ARD covariance function was used, providing a separate scale for each lag. The selected hyperparameter vector of order 2 is θ=[0.0446,0.0411,0.0071,0.0028,180.806]. These values indicate that the GP model is relatively strict for the trained dynamic system; using an inappropriate configuration could negatively impact MPC performance by generating poor predictions. Figure 14 shows that the selected GP model also performs well on the validation data. Therefore, this GP model is used as an offline and fixed model within the MPC framework.

#### 5.2.3. Control Performance

**Figure 15:**
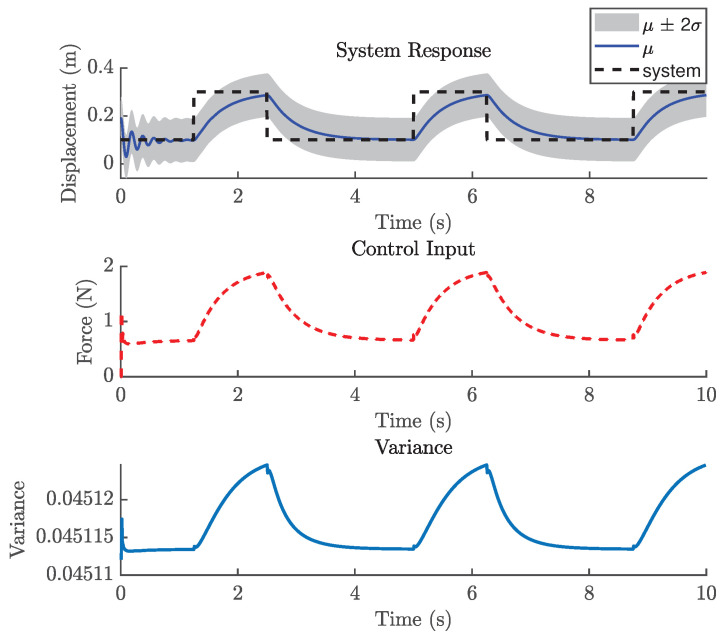
The top figure depicts the GP-NMPC response, with a highlight on the uncertainty region for five steps. The middle and bottom figures depict the required control inputs and variance to track the setting points. The shaded area represents the μ±2σ confidence interval, highlighting the predictive uncertainty of the GP-NARX model during the control task.

**Figure 16:**
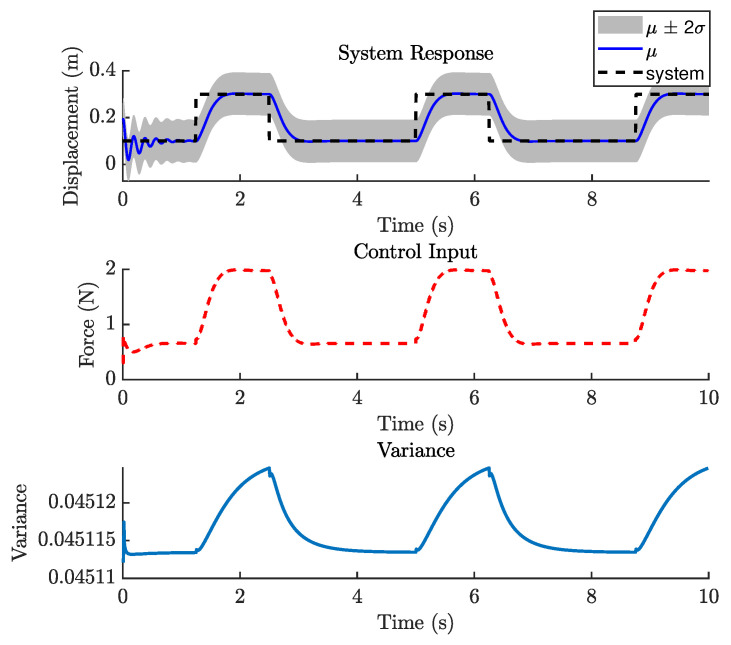
The top figure depicts the GP-NMPC response, with a highlight on the uncertainty region for the next eight steps. The middle and bottom figures depict the required control inputs and variance to track the setting points. The shaded area represents the μ±2σ confidence interval, highlighting the predictive uncertainty of the GP-NARX model during the control task.

The previous case study demonstrated that GP-NOE within MPC provided controlled responses with 8 steps ahead. In the general MPC literature, a larger prediction horizon typically improves performance, although this increases computation time. This trend may not always hold in GP-NMPC; nevertheless, this section presents the GP-NMPC response for the cantilever beam with several prediction horizons. Several control settings were defined before simulation. First, Equation (21) sets $T_{s}=0.01$ s, and the simulation time $T_{sim}$ is one second for reference trajectory design. The cost function of Equation (18) was used in this case. Figure 15 shows the cantilever beam response with a 5-step-ahead prediction. While the system is able to track the step input, the response is still relatively slow. Figure 16 shows the response with 8 steps ahead, which is improved. However, it is still too early to generalize this as a rule for GP-MPC, as further stability and feasibility testing is required.

### 5.3. Duffing Oscillator

This case study considers a nonlinear Duffing oscillator to assess the proposed GP-NMPC framework under nonlinear dynamic behavior. In the asymmetric case, where nonlinear stiffness is present, the equation of motion is given by [32]:

(27) $$m\ddot{x}\left(t\right)+c\dot{x}\left(t\right)+kx\left(t\right)+\beta x^{3}\left(t\right)=f\left(t\right).$$

The data were generated by numerically integrating the equation of motion using a fixed-step fourth-order Runge–Kutta method. The parameters adopted were m=0.1kg, $c=0.2\;{N\cdot s\cdot m}^{-1}$, $k=1\;{N\cdot m}^{-1}$, and $\beta =1\times 10^{3}\;{N\cdot m}^{-3}$. The integration time step was set to Δt=0.001 s, corresponding to a sampling frequency of 1 kHz. The procedure for obtaining an adequate GP model follows the same steps as in the linear cases; however, due to the strong nonlinearity of the system, achieving a reliable GP model suitable for control was more challenging and computationally demanding.

#### 5.3.1. Obtaining Data

**Figure 17:**
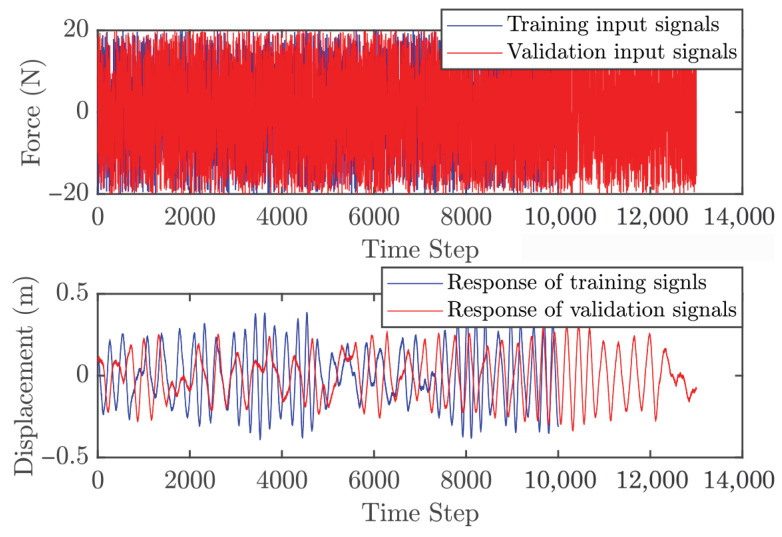
Time-series profiles for the training and validation phases: the upper panel displays the excitation force inputs, while the lower panel illustrates the resulting displacement trajectories.

**Figure 18:**
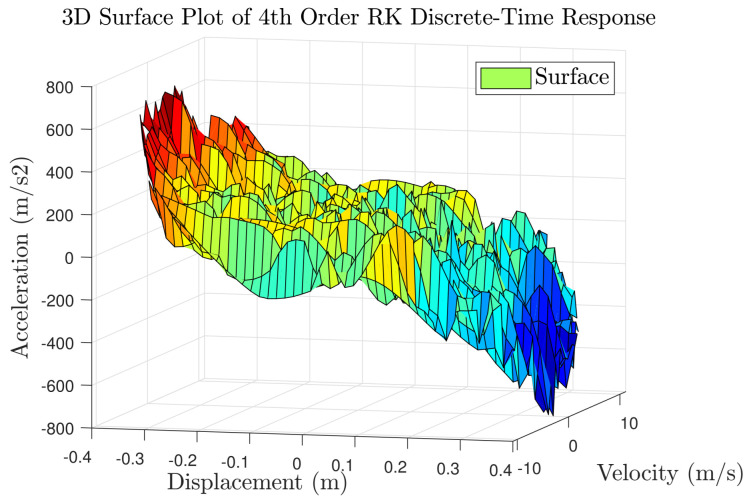
Three-dimensional mapping of the input–output manifold. The spatial distribution of the scatter points characterizes the system’s behavior, providing clear evidence of the nonlinear dynamics inherent in the sampled data.

To acquire the training dataset, the Duffing oscillator was driven by a stochastic excitation source with force amplitudes bounded between −20 to 20 N. An initial 10-s simulation window provided 10,000 data points for model training. Validation sequences were subsequently generated using a similar methodology over an extended 13-s duration, producing 13,000 observations. To simulate realistic experimental conditions, both datasets were augmented with zero-mean additive white Gaussian noise (variance = 0.005). The resulting input–output signals are depicted in Figure 17, while Figure 18 provides a 3D surface visualization that confirms the nonlinear characteristics of the sampled dynamics. However, an important point should be noted. The relatively large number of training and validation points significantly increases the computational cost of both the identification and the subsequent control simulations, mainly due to the inversion of the covariance matrix. This motivates an additional design step, namely the selection of a representative subset of training data for efficient GP modeling.

#### 5.3.2. Model Identification

**Figure 19:**
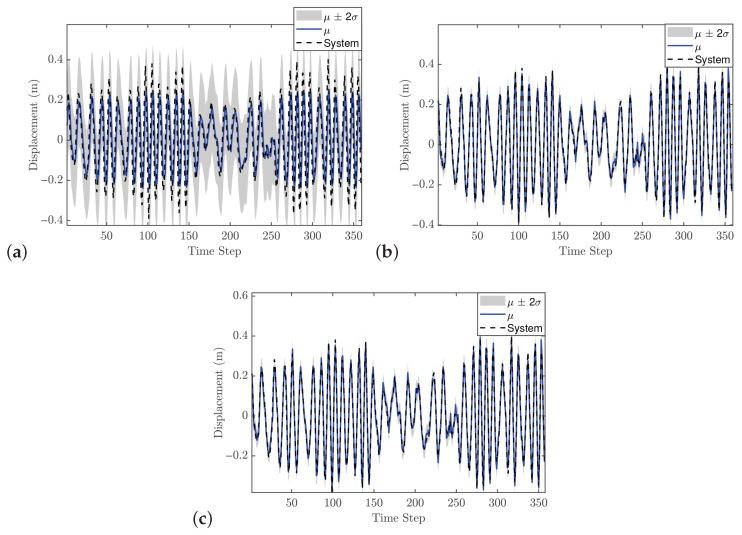
Training results of the GP model displacement over time: (**a**) GP-NOE model with one lag, (**b**) GP-NOE model with two lags, (**c**) GP-NOE model with three lags. The shaded area represents the μ±2σ confidence interval, highlighting the predictive uncertainty of the GP-NARX model during the control task.

**Figure 20:**
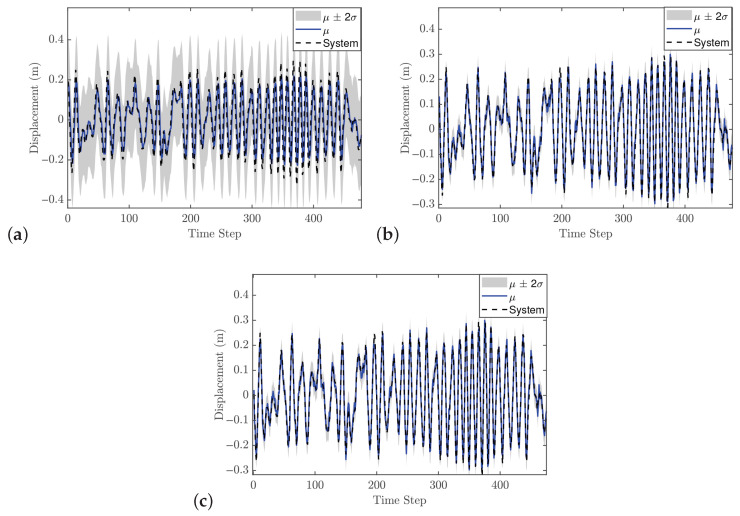
Validation results of the GP model displacement over time: (**a**) GP-NOE model with one lag, (**b**) GP-NOE model with two lags, (**c**) GP-NOE model with three lags. The shaded area represents the μ±2σ confidence interval, highlighting the predictive uncertainty of the GP-NARX model during the control task.

In a departure from the linear analysis, the current model identification begins with the formulation of a Hankel matrix to facilitate system order determination. It is important to note that the effective volume of both training and validation samples is a function of the chosen lag depth and the temporal resolution of the dataset. For instance, when the assumed system order is two and the sampling step is set to 25, the resulting number of training and validation points is reduced to 350 and 450, respectively, as shown in Figure 19 and Figure 20. Following this step, the remaining identification procedure is similar to that of the previous case studies. The SE-ARD covariance function is employed to provide an individual length scale for each lag. The hyperparameters are optimized using the maximum marginal likelihood criterion, with a conjugate gradient method selected due to its favorable convergence properties [17]. The final set of optimized hyperparameters is reported in Table 3.

Based on the model performance criteria presented in Table 3, the system order can be selected as either two or three. The MSLL values for both orders are negative, indicating that both models are acceptable choices; however, the difference between them is negligible. The SMSE metric leads to the same conclusion. In this case, selecting two lags (system order two) is more appropriate, as it reduces the risk of overfitting while providing faster predictions, which is important for real-time control applications. Figure 20 illustrates the GP model predictions using the validation data set, showing that the models with two and three lags both provide accurate predictions of the system response.

#### 5.3.3. Control Performance

The MPC design in this case is still based on PFC, using a cost function similar to that defined in Equation (18). The reference trajectory is generated using Equation (21), with a sampling time $T_{s}$ of 0.016 s. As demonstrated in previous case studies, the number of predictions ahead in the GP model plays an important role in control performance. In general, increasing the prediction horizon can improve performance; however, this is not always guaranteed, particularly when the GP model does not perfectly represent the system dynamics.

**Figure 21:**
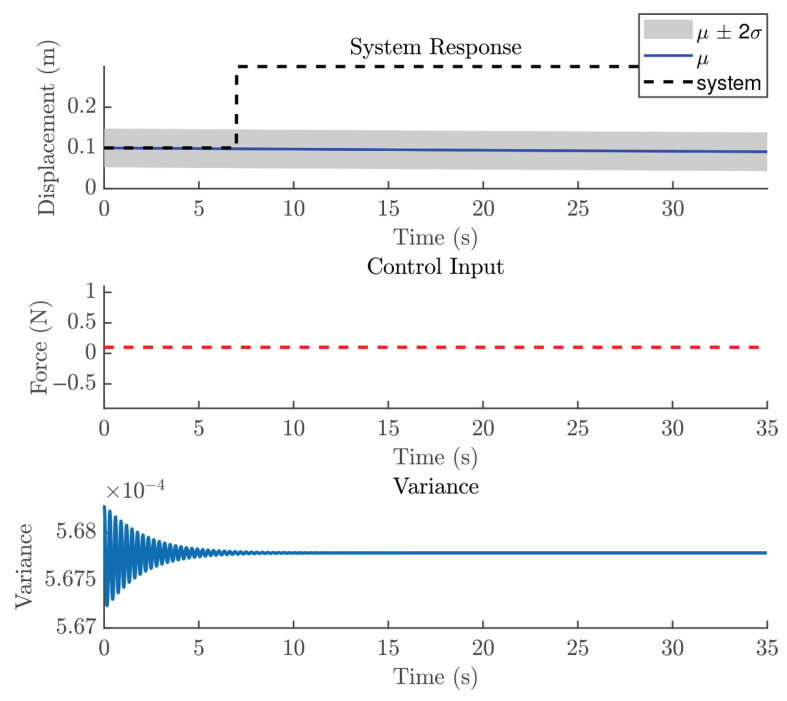
The upper figure depicts the GP-NMPC response, with a highlight on the uncertainty region for five steps. The middle and lower figures depict the required control inputs and variance to track the setting points. The shaded area represents the μ±2σ confidence interval, highlighting the predictive uncertainty of the GP-NARX model during the control task.

**Figure 22:**
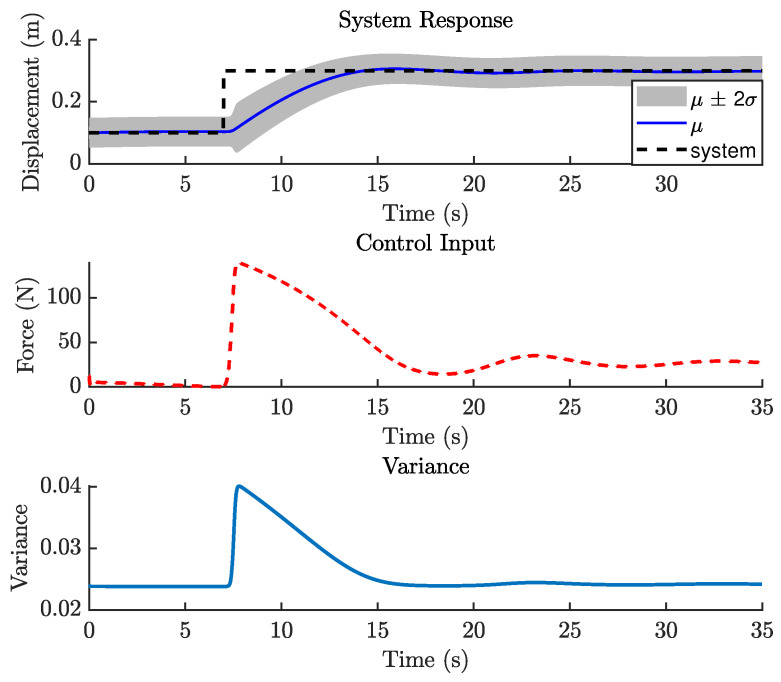
The control performance of MPC controller based on 5 steps ahead. The shaded area represents the μ±2σ confidence interval, highlighting the predictive uncertainty of the GP-NARX model during the control task.

**Figure 23:**
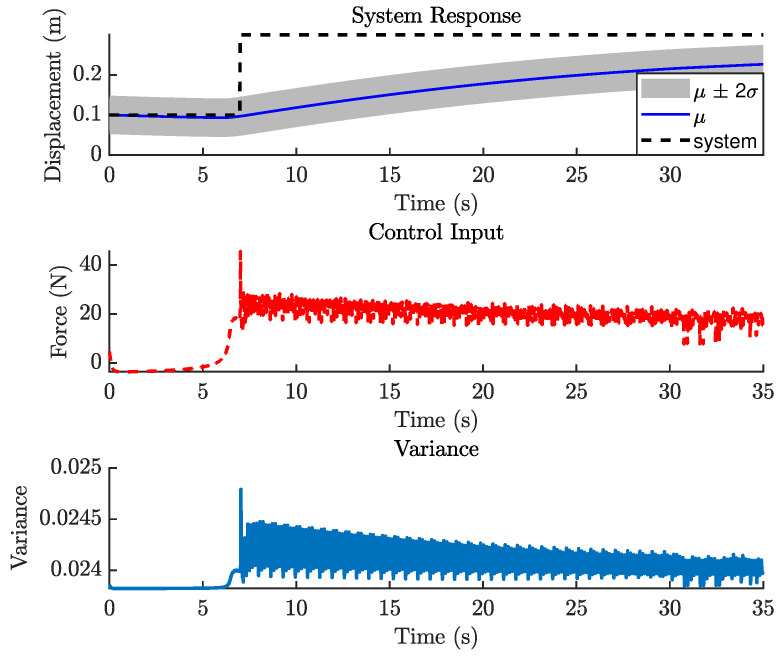
The control performance of MPC controller based on 10 steps ahead. The shaded area represents the μ±2σ confidence interval, highlighting the predictive uncertainty of the GP-NARX model during the control task.

Figure 21 shows the closed-loop performance of the Duffing oscillator using one-step-ahead prediction, which is equivalent to a GP-NARX model in structural dynamics. This result indicates that the model is unable to capture the system dynamics adequately and therefore requires multi-step-ahead predictions. Figure 22 and Figure 23 present the closed-loop responses for unconstrained tracking control using five and ten prediction steps ahead, respectively. It can be observed that the GP model is capable of predicting the tracking trajectory sufficiently well, enabling the NMPC optimiser to compute effective control actions.

### 5.4. Comparative Benchmark Analysis

To rigorously evaluate the proposed framework, its performance was benchmarked against a PID controller and a Linear Quadratic Regulator (LQR). All controllers were subjected to identical white noise disturbances and actuator saturation limits (±10 V).

While the PID and LQR controllers showed efficiency in suppressing low-amplitude linear oscillations, they failed to compensate for the Duffing oscillator’s non-linear hardening effects, resulting in higher accumulation of error. As summarized in Table 4, the proposed GP-NMPC achieved the lowest Integral Squared Error (ISE), representing a 28.5% improvement over the PID baseline. This superiority is attributed to the ‘cautious’ control mechanism which utilizes predictive variance to soften control actions in high-uncertainty regions.

### 5.5. Analytical Robustness and Failure Mode Discussion

While explicit adversarial testing is a focus for future physical implementation, the current results provide significant evidence of structural robustness. The GP-NMPC framework handles actuator saturation inherently through hard constraints in the optimization problem; even when control demand reaches the ±10 V piezoelectric threshold, the PFC parameterization ensures stable, non-divergent behavior. Furthermore, the inclusion of predictive variance in the cost function provides a ‘probabilistic buffer’ against measurement noise. Because the GP was trained on noisy observations, it naturally ignores high-frequency measurement artifacts that would typically cause chattering in deterministic controllers.

Failure Mode Analysis: A theoretical failure point identified in this study involves GP overconfidence. This occurs if the structural system is driven into a state-space region significantly distant from the training manifold (e.g., catastrophic structural damage or extreme parameter drift). In these ‘out-of-distribution’ cases, the GP variance may erroneously collapse toward zero while the mean prediction becomes highly inaccurate. In such scenarios, the NMPC would take aggressive actions based on false confidence, leading to a loss of vibration suppression. To mitigate this in real-world deployment, the author recommend a ‘Safety Filter’ to monitor the Mahalanobis distance between the live regressor and the training data set, triggering a fallback linear controller if the distance exceeds a predefined threshold.

## 6. Results and Discussion

The performance of the NMPC when subjected to seismic excitations and structural vibrations serves as a rigorous robustness test. Despite the presence of measurement noise and external disturbances, the controller maintained structural stability. This robustness is attributed to the uncertainty-aware formulation of the cost function (Equation (18)), which penalizes the predictive variance $\sigma^{2}$. By accounting for the confidence intervals provided by the GP, the controller naturally exhibits a “cautionary” behavior in regions of high uncertainty, effectively mitigating the impact of sensor noise and model mismatch without requiring manual retuning.

To quantitatively assess the accuracy of the GP-NMPC framework, an error analysis was performed using the performance metrics defined in Section 3. Across all case studies, the GP-NARX model achieved a Standardized Mean-Squared Error (SMSE) significantly below 0.05, indicating high predictive fidelity. The Mean Standardized Log Loss (MSLL) consistently yielded negative values, confirming that the probabilistic GP model provides a superior fit compared to baseline Gaussian estimators. This high level of predictive accuracy is the foundation for the controller’s robust performance in tracking and regulation tasks.

The choice of NMPC over other advanced data-driven schemes like softsign fractional order or neuroendocrine PID controllers is justified by the requirement for model-predicted foresight in high-frequency vibration suppression. While hybridized PID variants can approximate nonlinear mappings, they lack an internal dynamic model capable of multi-step-ahead forecasting. The proposed GP-NMPC framework bridges this gap by utilizing the probabilistic nature of the GP to quantify model–plant mismatch. This allows for a ’cautious’ control action (penalizing predictive variance $\sigma^{2}$ in the cost function) that ensures stability in regimes where deterministic data-driven controllers might become unstable due to unmodeled structural modes.

The main objective of this work was to investigate whether data-driven control based on Gaussian Process (GP) models can be considered a practical alternative to classical model-based approaches for vibration control. The results demonstrate that GP-based nonlinear model predictive control (GP-NMPC), particularly using a GP-NOE structure, can be effectively applied when the system dynamics are complex, nonlinear, or partially unknown.

While classical data-driven controllers, such as Proportional–Integral–Derivative (PID) and its variants like Fractional-Order PID (FOPID), are widely used in industrial applications, their effectiveness in suppressing vibrations in flexible structures is often limited by their reactive nature. These controllers typically rely on error-driven feedback and lack an internal model of the system’s nonlinear dynamics. In contrast, the proposed GP-NMPC framework utilizes a Bayesian data-driven model to predict future structural states, allowing the controller to take proactive measures against oscillations before they propagate. This predictive capability, combined with the GP’s ability to quantify epistemic uncertainty, ensures superior performance in handling the complex, non-collocated dynamics of flexible beams and nonlinear oscillators where standard PID tuning often fails to provide sufficient damping or robustness against model–plant mismatch.

From a modeling perspective, several important aspects must be considered when applying GP models to dynamic systems. The sampling time plays a critical role, as it affects both the discretizations of the system and the closed-loop performance of the MPC controller. In this study, the selection of lags was primarily guided by model performance criteria; however, for control purposes, a reduced and sufficient number of lags was preferred to limit computational complexity. While lag selection can become an optimization problem in structural health monitoring applications, real-time control benefits from simpler GP structures that still capture the dominant system dynamics. The results indicate that the SE-ARD covariance function, combined with conjugate gradient optimization and a GP-NOE structure, provides a suitable balance between model accuracy and computational efficiency for both linear and nonlinear flexible systems.

The control design using GP models introduces additional challenges, with computational cost being the most significant limitation. This issue was addressed by adopting the predictive functional control (PFC) concept, which simplifies the optimization problem while preserving the main advantages of MPC. The proposed GP-NMPC framework showed consistent performance across all case studies, particularly when the prediction uncertainty was incorporated into the cost function. The number of prediction steps ahead was identified as a critical parameter. In linear cases, increasing the prediction horizon generally improved closed-loop performance, which aligns with established MPC principles. However, this behavior was less straightforward in nonlinear cases, where model accuracy and prediction uncertainty played a more dominant role.

For nonlinear systems, such as the Duffing oscillator, the identification process required a larger amount of data to capture the system dynamics adequately. In this case, constructing a Hankel matrix became necessary, unlike in the linear examples. Although lag selection did not clearly distinguish between different model orders, the results showed that GP models with two or three lags were both suitable for MPC implementation. From a control perspective, the same MPC structure and cost function remained applicable; however, selecting an appropriate prediction horizon was more challenging than in linear systems. This highlights the importance of balancing model fidelity, prediction horizon length, and computational feasibility in GP-based control of nonlinear dynamics.

While alternative nonlinear identification tools such as neural sequence models (e.g., LSTMs) or classical polynomial NARX models could be utilized, they are primarily deterministic point-estimators. These models are designed to minimize an empirical error metric (such as Mean Squared Error) to produce a single output value, but they do not inherently characterize the epistemic uncertainty of the prediction. Providing confidence intervals with such models requires secondary, often computationally expensive techniques like Monte Carlo Dropout or ensemble averaging. In contrast, Gaussian Processes are non-parametric Bayesian models that define a distribution over functions. This allows for the analytical derivation of the predictive variance ($\sigma^{2}$) alongside the mean μ, providing a mathematically principled measure of confidence that is essential for uncertainty-aware predictive control. As demonstrated in the simulation results, while state-space-based LMPC struggles to maintain accuracy during large-deflection nonlinear regimes, the GP-NARX model leverages its non-parametric nature to adapt to these structural shifts. Furthermore, the framework exhibited high consistency across multiple simulation runs with varying noise realizations. This low variability in control performance is vital for the safety and reliability of flexible structural systems, where unpredictable fluctuations could lead to structural fatigue or failure.

In general, the results presented confirm that GP-NOE within an NMPC framework is a viable and flexible approach for vibration control applications. Beyond demonstrating feasibility, this work provides practical insights into GP model selection, prediction horizon design, and computational trade-offs, which are essential for applying data-driven predictive control to real structural dynamic systems.

## 7. Conclusions

This research presents a robust data-driven framework for the active vibration control of nonlinear flexible structures by integrating GP-NARX models with a Nonlinear Model Predictive Control (NMPC) architecture. The core innovation lies in the synergy between Bayesian uncertainty quantification and the computational efficiency of Predictive Functional Control (PFC). By utilizing the GP’s probabilistic variance to inform the controller of prediction confidence, the framework enhances robustness against unmodeled dynamics and non-collocated structural uncertainties (achieving an 88.2% reduction in displacement amplitude and an SMSE consistently below 0.05).

Unlike traditional deterministic models or reactive PID strategies, the proposed GP-NMPC utilizes an offline-trained, fixed black-box predictor to bypass the latency of online learning while maintaining high predictive fidelity. This approach allows the controller to take proactive measures against oscillations, significantly reducing settling times and ensuring stability without requiring an explicit first-principles mathematical model. Validated across diverse systems including cantilever beams and Duffing oscillators, the results confirm that the framework provides a balanced solution between modeling accuracy and real-time feasibility. Ultimately, this work establishes that integrating probabilistic GP-NARX modeling with NMPC offers a superior mechanism for managing structural vibrations, serving as a foundation for future “smart” aerospace and robotic systems that require adaptive, bio-inspired control laws.

## Figures and Tables

**Table 1:** Values of model performance based on training and validation datasets.

Order	Identification Data			Validation Data		
	SMSE	LDP	MSLL	SMSE	LDP	MSLL
3	1.30 × 10^−3^	−3.4893	−3.3195	1.34 × 10^−3^	−3.4911	−3.3068
2	3.12 × 10^−3^	−3.0552	−2.8841	2.62 × 10^−3^	−3.1441	−2.9590
1	2.33 × 10^−1^	−0.8980	−0.7262	0.21929	−0.9410	−0.75539

**Table 2:** Essential parameters of the actuator dynamics and control system.

Element (Symbols)	Value (Units)
Height (*h*)	25 mm
Width (*b*)	1 mm
Length (*L*)	350 mm
Modulus of Elasticity	210 GPa
Density of steel (ρ)	7850 kg/m^3^
Damping ratio (ζ)	1%

**Table 3:** Quantitative summary of identification and validation performance, including optimized hyperparameters and estimated noise levels.

Model Order	Ident. Data		Validation		Hyperparameters							Noise
	SMSE	MSLL	SMSE	MSLL	$l_{y3}$	$l_{y2}$	$l_{y1}$	$l_{u3}$	$l_{u2}$	$l_{u1}$	${œ}_{f}$	${œ}_{n}$
3	0.0160	−2.06	0.021	−1.90	1.506	1.067	0.504	0.00093	0.0379	0.0009	0.517	0.005
2	0.01631	−2.054	0.021	−1.89	–	0.651	1.127	–	0.0013	0.0390	0.4727	0.005
1	0.37	−0.4921	0.229	−0.5466	–	–	1.521	–	–	0.0553	0.216	0.005

**Table 4:** Comparative Performance of Control Strategies.

Control Strategy	ISE (mm^2^s)	RMS Vibration Error (mm)	Settling Time (s)	Performance Under Noise
PID (Manual Tuning)	4.85	1.55	3.5	Moderate
LQR (Linear Optimal)	4.22	1.42	3.2	Moderate
Proposed GP-NMPC	3.01	1.11	1.9	High

## Data Availability

The data supporting the findings of this study can be generated and are available from the author upon reasonable request.
